# miR-340: A multifunctional role in human malignant diseases

**DOI:** 10.7150/ijbs.51123

**Published:** 2021-01-01

**Authors:** Zheng Huang, Yesha Xu, Maoping Wan, Xixi Zeng, Jianmin Wu

**Affiliations:** 1 Institute of Genomic Medicine, Wenzhou Medical University, Wenzhou 325035, Zhejiang, P.R. China.; 2 Department of Anesthesia and Intensive Care, The Chinese University of Hong Kong, Hong Kong Special Administrative Region, P.R. China.

**Keywords:** microRNAs, miR-340, malignant diseases, cancers, drug resistance

## Abstract

MicroRNAs (miRNAs) are a class of short non-coding RNAs of approximately 22 nucleotides in length, which function by binding to the 3' UTR sequences of their target mRNAs. It has been reported that dysregulated miRNAs play pivotal roles in numerous diseases, including cancers, such as gastric, breast, colorectal, ovarian, and other cancers. Recent research efforts have been devoted to translating these basic discoveries into clinical applications that could improve the therapeutic outcome in patients with cancer. Early studies have shown that miR-340 may act either as an oncogene or a tumor suppressor by targeting genes related to proliferation, apoptosis, and metastasis, as well as those associated with diagnosis, treatment, chemoresistance, and prognosis. miR-340 has been shown to have a role in other diseases, such as autoimmune diseases, acute stroke, and alcoholic steatohepatitis. Nevertheless, the roles of miR-340 in human malignancies are still unclear, and the associated mechanisms are complex, involving a variety of signaling pathways, such as Wnt/β-catenin and the JAK-STAT pathways. Herein, we review the crucial roles of miR-340 in human cancers through the analysis of the latest research studies, with the aim of clarifying miR-340 function in malignant disease diagnosis, treatment, and prognosis, and to propose further investigations.

## Introduction

Cancer is a major public health problem worldwide. The GLOBOCAN 2018 estimated that there would be 18.1 million new cases and 9.6 million deaths from cancer in 2018 1. Siegel et al. reported that there might be 1,762,450 new cancer cases and 606,880 cancer-related deaths projected to occur in the United States in 2019 2. In China, Chen et al. predicted that there would be about 4,292,000 newly diagnosed invasive cancer cases in 2015, based on the data collected from 72 cancer registries 3. Therefore, it is imperative to investigate the correlation between cancers and their respective risk factors; it is especially important to understand the molecular mechanisms of cancers, which may contribute to developing novel and effective pharmaceuticals and treatments.

MicroRNAs (miRNAs) are small non-coding RNAs of about 22 nucleotides in length. The mature miRNA is loaded into the RNA-induced silencing complex (RISC), which is directed to target mRNAs, leading to translational repression and target mRNA degradation 4. Recently, numerous studies have reported the roles for miRNAs in several human malignant diseases, especially cancers. miRNAs can function as modulators in multiple biological and pathological processes, such as cancer cell differentiation, proliferation, and apoptosis 5. They can be used in cancer monitoring and therapy, and even in the clinical assessment of cancer patient outcomes 6. For instance, Wang et al. have revealed that serum exosomal miR-17-5p, miR-130a-3p, and miR-93a-5p were downregulated and associated with breast cancer (BC) recurrence or distant organ metastasis 7. Conversely, several upregulated miRNAs promoted tumor development and malignancy in ovarian cancer (OC) and intrahepatic cholangiocarcinoma 8, 9. Moreover, miRNAs functioned as vital modulators in radiation-induced aggressive tumor behavior in human carcinoma cells 10. These findings have led to the clinical trials of miR-34 replacement therapy (NCT01829971; terminated due to severe immune-related adverse events) and miR-122-based therapy LNA-antimiR-122 (SPC3649; successfully undergoing phase II trials) in cancer patients 11, 12. Thus, we believe that miRNA-based therapy may be a novel and promising method with which to treat various tumors in the future, when the roles and mechanisms of miRNAs in cancer are clearly understood.

**Figure 1:**
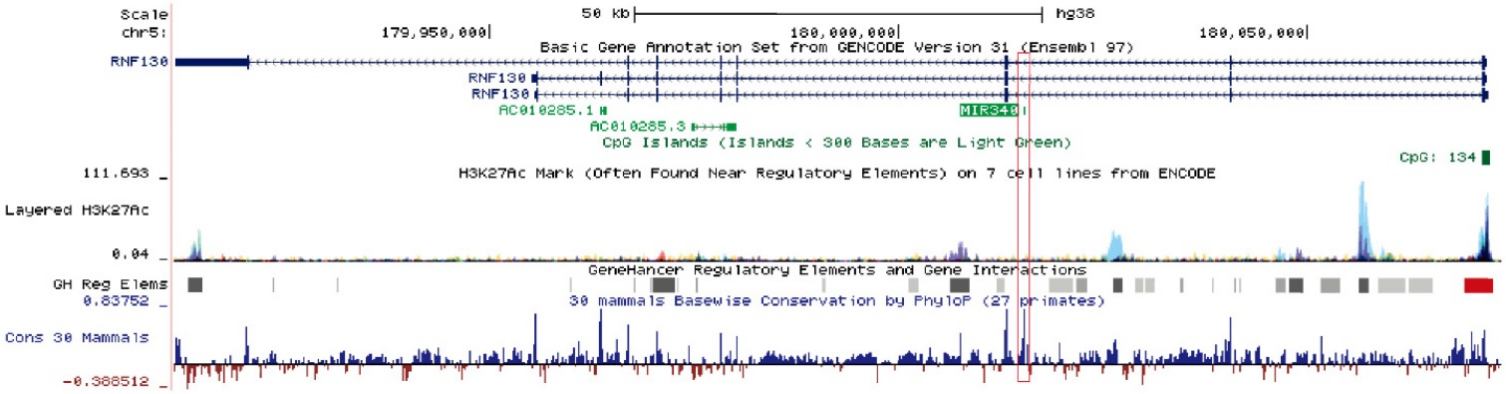
Schematic diagram of the miR-340 locus. miR-340 is an intragenic miRNA located in the intronic region of the host gene *RNF130.* It has similar expression patterns as those of the host gene. In the upstream regulatory regions as well as the gene body, an active epigenetic marker, the H3K27Ac cluster, was identified by the ENCODE project; a high-confidence enhancer/promoter cluster was identified by the GeneHancer project. The information was obtained from the UCSC Genome Browser.

Human miR-340 is an intragenic miRNA, located in the intronic region of the host gene *RNF130*, on chromosome 5q35.3 13. miR-340 is highly conserved among mammals, and its expression pattern is similar to that of the host gene. One study showed that miR-340-5p expression correlated with that of *RNF130* in myeloma cell lines, and the expression of miR-340-5p was regulated by promoter hypermethylation of *RNF130* 13. The clustered H3K27Ac and high-confidence regulatory elements have been identified in the upstream region of miR-340, suggesting that the miR-340 locus was actively transcribed and intricately regulated (**Fig. 1**). miR-340 has been shown to participate in the progression of several diseases. Serum miR-340-5p levels were shown to be dysregulated in pulmonary sarcoidosis with Lofgren's syndrome 14, and serum miR-340-3p acted as a genetic biomarker associated with human longevity 15. miR-340-5p was involved not only in human heart failure and pathological cardiac hypertrophy 16, but also in osteoclast formation and osteoporosis progression 17. Recently, a number of studies have reported that miR-340 plays a critical role in tumor initiation and progression, by targeting multiple oncogenes such as *SKP2*, *FHL2*, *c-Met*, and *ROCK1* (**Table 1**). Some signaling pathways related to tumorigenesis, such as AKT 18, SOCS3/JAK-STAT 19, and Wnt/β-catenin pathways 20, were shown to be suppressed by miR-340.

In this review, we present and synthesize the roles and mechanisms of action of miR-340 in cancer cell proliferation, apoptosis, invasion and metastasis, drug resistance, and cancer diagnosis, as well as patient survival and prognosis, aiming to provide a significant foundation for clinical applications and future investigations.

## Biogenesis and expression regulation of miR-340

Previous reports have demonstrated that the gene promoter region, which is distal to the transcription start site, contains uniformly dense CpG islands. These islands were methylated in cancer cells, but not in normal cells 21. Transcriptional silencing via CpG methylation in the promoter is an important regulatory mechanism for the downregulation of miRNAs, which may promote tumor initiation and development 20, 22-25. Recently, hypermethylation of CpG islands in the miR-340 gene promoter region has been found in gastric cancer (GC) 26. Our recent study revealed that treatment with 5-aza-2'-deoxycytidine or TSA restored the levels of miR-340 in OC cells 20. These results implied that the silencing of miR-340 expression was mediated through the CpG methylation of the upstream regions in the miR-340 promoter.

Transcription factors act as regulators that modulate miRNA expression in many cancers. For example, a decrease in SIRT1 stimulates the expression of p53, which leads to the activation of miR-34a 27, 28. Similarly, zinc finger E-box-binding homeobox 1 (ZEB1) acts as a transcription factor that suppresses miR-340 expression in BC 29. Hypoxia is a common microenvironment in multi-pathophysiologic progression, including tumorigenesis 30, 31. There is increasing evidence that miRNAs are involved in tumorigenesis and drug resistance driven by hypoxia 32, 33. miR-340-5p was identified as being significantly downregulated by hypoxia in melanoma cells 34. Du et al. found that the antioxidant ferulic acid could promote hypoxia signaling by inducing hypoxic-induced factor, which suppressed miR-340-5p promoter activation through hypoxia response element (HRE) motifs 35.

**Figure 2:**
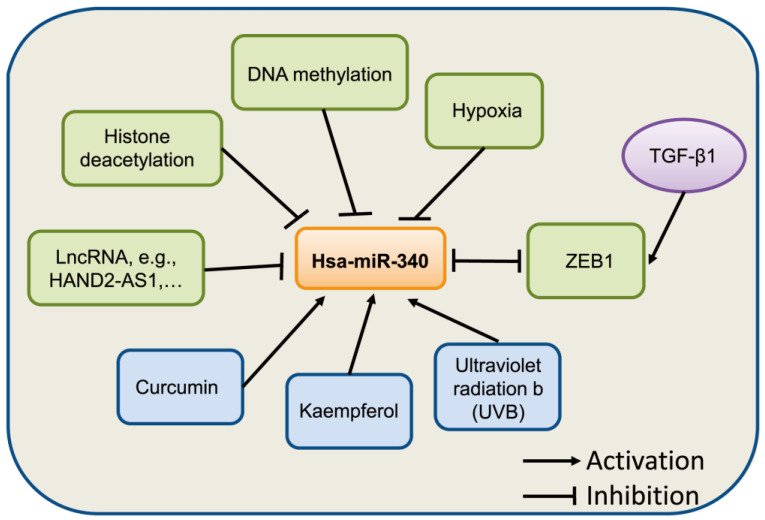
Summary of miR-340 expression regulation. miR-340 expression is modulated by various factors, such as epigenetic modification, transcription factors, and hypoxia.

The natural compound curcumin has also been shown to induce the expression of miR-340 in human pancreatic cancer cells. miR-340 was identified as being significantly upregulated following curcumin treatment 36. A recent study showed that miR-340 is regulated by nanocurcumin in relapsing-remitting multiple sclerosis 37. Another compound, Kaempferol, was found to upregulate miR-340 expression in human lung cancer cells 38. In retinal pigment epithelium (RPE) cells 39, UVB irradiation induced the expression of miR-340, which promoted RPE cell apoptosis and suppressed cell viability by affecting p53, p21, and caspase-3 protein expression. Collectively, these evidences suggest that the expression of miR-340 can be regulated by epigenetic modification, transcriptional regulation, and other factors, although further studies on the mechanism underlying miR-340 dysregulation in human cancer are needed (**Fig. 2**).

## Functional roles of miR-340 in cancers

### Role of miR-340 in cell proliferation in cancer

Increasing evidence has indicated that miR-340 is associated with various genes mediating cancer cell proliferation. Cyclin D/cyclin G2 and CDK4/6 have been shown to be upregulated in a variety of human cancer cells 40-42. The p27/p21 gene, which is a cell cycle inhibitor, binds to and prevents the activation of cyclin E-CDK2 or cyclin D-CDK4/6 complexes, and thus controls cell cycle progression at the G1 phase, which is known to be involved in cancers 43. miR-340 was shown to induce the accumulation of p27 and subsequent cell cycle arrest by targeting three negative regulators of p27: PUM1, PUM2, and SKP2, indicating that miR-340 could repress non-small cell lung cancer (NSCLC) cell proliferation 44. miR-340 also inhibits lung cancer cell proliferation by targeting CDK4 40; while it has been reported that CDK4 is involved in accelerating NSCLC cancer procession and combined inhibition of CDK4 could be effective to treat NSCLC 45. Additionally, transfection of miR-340 or silencing of EZH2 has been shown to impede laryngeal squamous cell carcinoma progression by inducing p27 expression and suppressing PI3K/AKT activation 46.

In hepatocellular carcinoma (HCC), miR-340 inhibits cell proliferation and tumor growth by inhibiting SKP2 expression 47, and regulating the JAK1/STAT3 pathway 48. Additionally, miR-340 also functions as a tumor suppressor in colorectal cancer (CRC) by regulating the alternative splicing of the *PKM* gene 49 or directly targeting *RLIP76* 50. Restoration of miR-340 expression in angiosarcoma cells reduced cell proliferation by negatively regulating *SIRT7* expression 51. In addition, miR-340 expression was discovered to be downregulated in both glioma cell lines and tissues. Increasing miR-340 levels dramatically inhibited glioma cell proliferation, and induced cell-cycle arrest via inhibition of the target gene *ROCK1*, as well as several oncogenes, including p*-AKT*, *EZH2*, *EGFR*, *BMI1*, and *XIAP* 52. miR-340 was found to specifically target the 3' UTRs of *CDK6*, cyclin-D1, and cyclin-D2, leading to the arrest of glioblastoma multiforme (GBM) cells in the G0/G1 cell cycle phase 42. Furthermore, miR-340 was shown to be a potential therapeutic agent for GBM via its inhibitory effects on Bcl-w-induced platelet-derived growth factor-A (PDGF-A) and Sox2 activation 53. A recent study revealed that miR-340 could suppress osteosarcoma (OS) cell proliferation by inactivating the Notch signaling pathway by down-regulating *CTNNB1* 54. It has been reported that the overexpression of miR-340 inhibited human esophageal squamous cell carcinoma (ESCC) cell growth by modulating the expression of phosphoserine aminotransferase 1 (*PSAT1*), and it may contribute to the progression of ESCC 55. Additionally, miR-340 inhibited BC cell proliferation by targeting the expression of *ZEB1* or *LGR5* through the Wnt/β-catenin pathway, which might provide a new perspective for BC treatment 29, 56. miR-340 functioned as a tumor suppressor in prostate cancer (PCa) through the MDM2-p53 pathway by directly targeting *MDM2* 57.

Nevertheless, Xiao and colleagues found that the level of miR-340 was significantly higher in MKN-28 cells than in GES-1 cells; anti-miR-340 attenuated cell proliferation and arrested cell cycle in MKN-28 cells by upregulating *SOCS3* expression to suppress the JAK-STAT3 signaling pathway 19. miR-340 was found to promote thyroid cancer growth *in vitro* and *in vivo* by inhibiting *BMP4* 58. The role of miR-340 is ambiguous in GC 19, 59, and needs to be further investigated.

**Figure 3:**
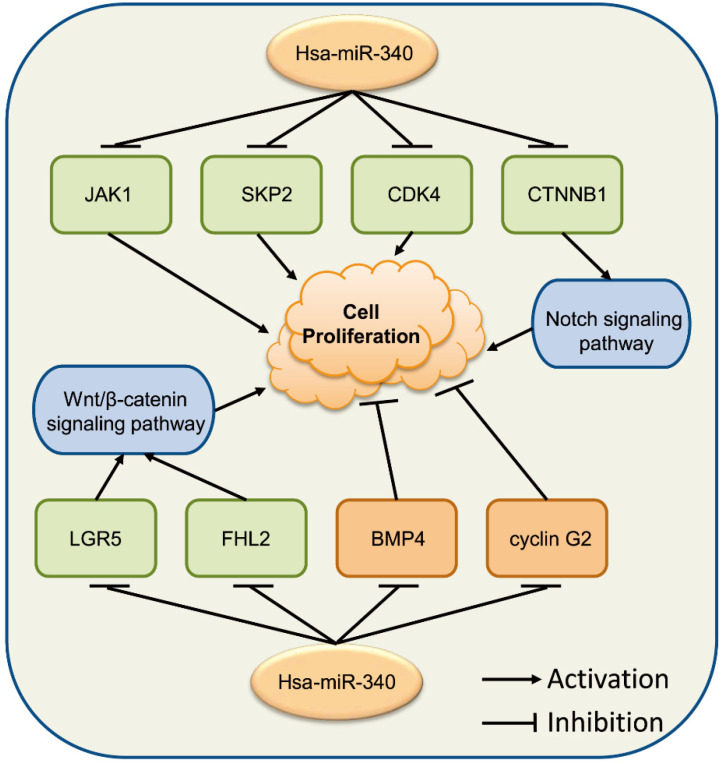
miR-340 is associated with various genes modulating cancer cell proliferation. Upregulated miR-340 inhibits the expression of *JAK1, SKP2*, and *CDK4*, repressing cell proliferation, while it inhibits the expression of *BMP4* and cyclin G2, inducing cell proliferation. miR-340 inhibits the Wnt/β-catenin signaling pathway by targeting *LGR5* or *FHL2*, as well as the CTNNB1-mediated Notch signaling pathway, resulting in repressed cell proliferation.

These observations show that the relationship between miR-340 and cancer cell progression is complex (**Fig. 3**). miR-340 might perform different context-dependent roles in different cancer cells, acting as either a tumor growth suppressor or an oncogene, which requires further investigation to verify its exact roles in specific cancers.

### miR-340 and cell apoptosis

**Figure 4:**
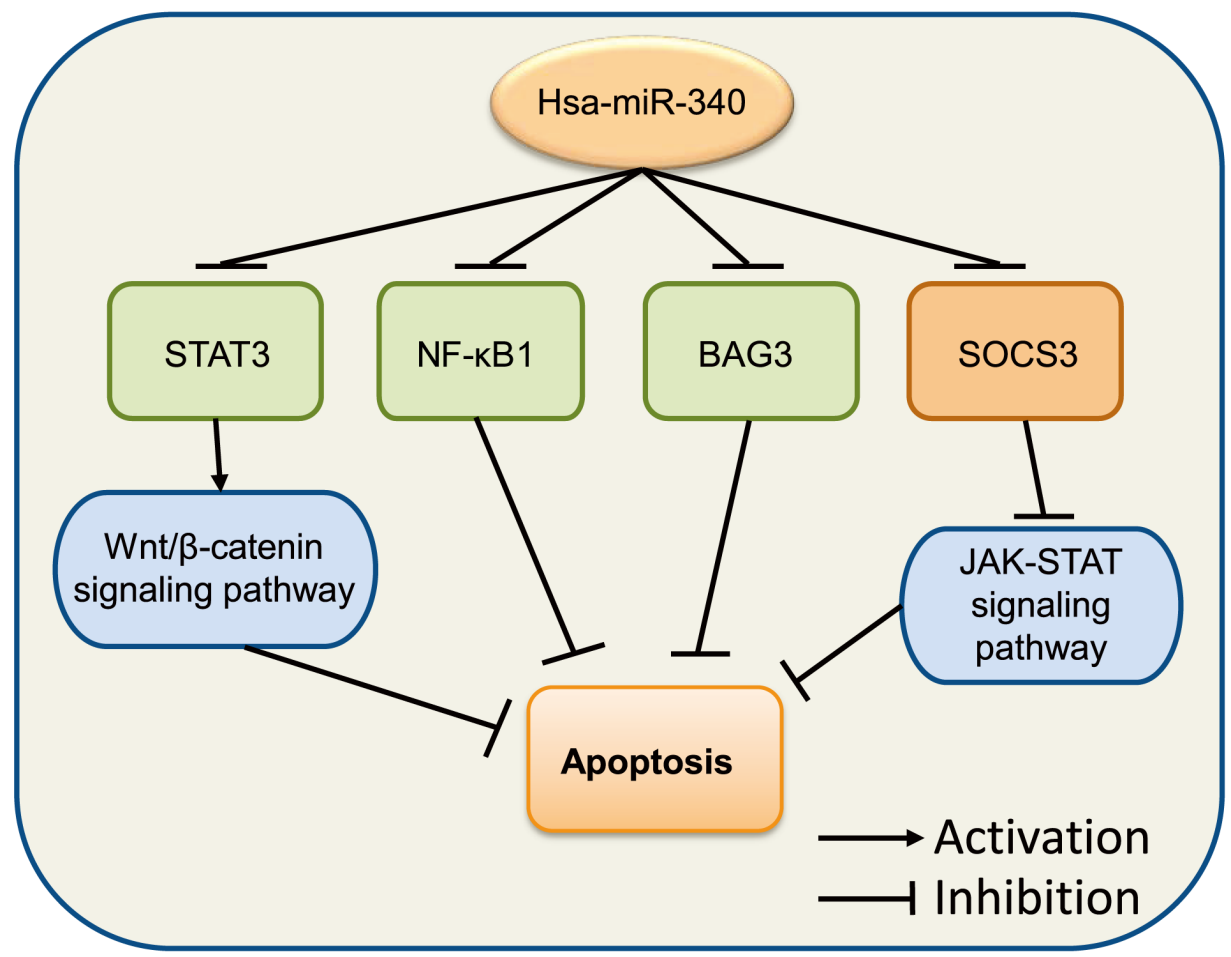
miR-340 is involved in the regulation of cancer cell apoptosis. Upregulated miR-340 inhibits NF-κB1, BAG3, and STATS as well as its downstream Wnt/β-catenin signaling pathway, which induces cell apoptosis, while it inhibits the expression of SOCS3 but suppresses apoptosis by regulating the JAK-STAT signaling pathway.

Accumulating evidence suggests that the dysregulation of cell apoptosis is involved in a majority of diseases, as this process includes multitudes of classical signaling pathways and proteins. Death receptors, mitochondria, and caspase signaling pathways have been reported to participate in cancer modulation 60. Bcl-2 and Bax are members of the Bcl-2 family, which has been implicated in the regulation of cell apoptosis 61. Bcl-2 is an anti-apoptotic protein and can directly prevent cell apoptosis by limiting the pro-apoptosis member activity of the Bcl-2 family 62. Bax is a proapoptotic member of the Bcl-2 family that regulates programmed cell death, and is associated with increased survival 63. miR-340 increased the expression levels of BIM and Bax, but decreased those of Notch and Bcl-2, inducing OS cell apoptosis by inactivating the Notch signaling pathway via targeting *CTNNB1* 54. In CRC, miR-340 was observed to target *RLIP76* 50 and *REV3L* 64 to mediate cell apoptosis. Moreover, miR-340 increased the levels of apoptosis-related factors pro-caspase 3, cleaved-caspase 3, and Bax, but inhibited Bcl-2 in SGC-7901 cells 18. A similar phenomenon was also observed in the endometrial carcinoma cell line RL 95-2 65. In OC, miR-340 was shown to induce cell apoptosis by the downregulation of NF-κB1 66. Overexpression of miR-340 improved apoptosis in SKOV3 cells through the negative regulation of BAG3, which might be involved in the regulation of the PI3K/AKT pathway 67. Altogether, these studies indicated that miR-340 exerts significant effects in cancer cell apoptosis (**Fig. 4**).

### Role of miR-340 in cancer invasion and metastasis

Cancer invasion and metastasis are usually happened in highly malignant cancer patients and involved in complex mechanism. The epithelial-mesenchymal transition (EMT) is a key process in tumorigenesis, and Vimentin and E-cadherin are typical biomarkers of EMT 68. Wu et al. performed microarray-based profiling analysis of miRNA expression in BC lines with different invasion capacity and found that miR-340 was significantly decreased in BC cell lines with high invasive potential 69, 70. Restoration of miR-340 in the BC cell line suppressed the expression of target genes, such as *c-Met* 70, *CTNNB1*, *c*-*MYC* 71, and *MYO10* (myosin X) 72 to inhibit cell invasion and metastasis via several signaling pathways. Transforming growth factor (TGF)-β signaling is important for EMT and the expression of *ZEB1* 73; treatment with the TGF-β1 resulted in increased levels of *ZEB1* expression, while decreasing the level of miR-340 in BC 29. Overexpression of miR-340 inhibited the migration and invasion of cervical cancer cells by targeting *EphA3* and adjusting the EMT pathway 74. Infection with the hepatitis B virus (HBV) is a leading cause of hepatocellular carcinoma (HCC) 75, and HBV promotes the migration of liver cancer cells by downregulating miR-340-5p to induce STAT3 overexpression, indicating that STAT3 plays a key role in regulating cell migration in HBV-HCC involving miR-340-5p 76. It has been observed that miR-340 suppressed tumor growth and metastasis in OS cells *in vitro* and *in vivo* by targeting *ROCK1* 77. Takeyama et al. found that miR-340 expression was significantly decreased in the EpCAM(+) bone marrow cells of CRC patients with liver metastasis, showing that miR-340 in the bone marrow might play an important role in regulating the metastasis cascade in CRC 78. In gallbladder carcinoma, miR-340 targeted *NT5E* to function as a significant suppressor of tumor metastasis 79.

**Figure 5:**
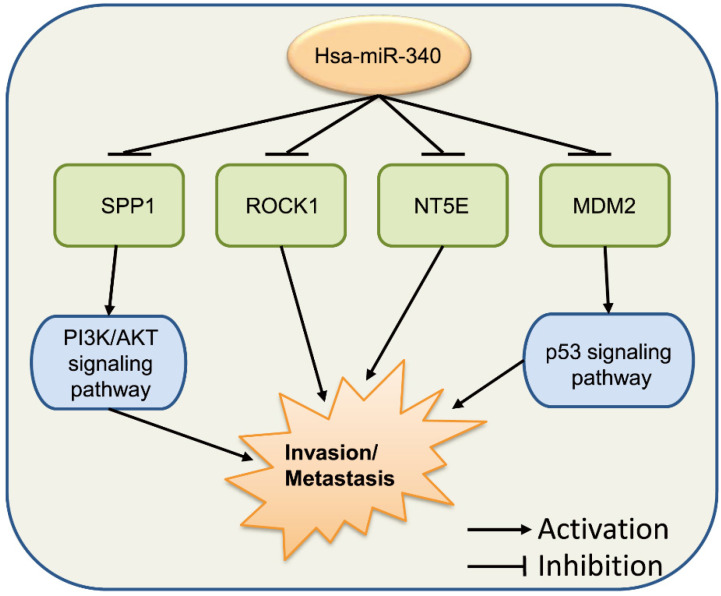
miR-340 functions in cancer invasion and metastasis. *ROCK1*, *NT5E*, *SPP1,* and *MDM2* are the target genes of miR-340, and miR-340 inhibits their functions as well as the downstream PI3K/AKT and p53 signaling pathways, which results in the repressed cell invasion and metastasis.

These series of findings indicate that miR-340 has vital functions in tumor invasion and metastasis (**Fig. 5**). Increasing the levels of miR-340 expression appears to repress cancer invasion and metastasis in most types of cancer. We hypothesize that these findings cover only a few functions of miR-340 in tumor invasion and metastasis. In addition, other targets and signaling pathways related to miR-340 might be included in further investigations.

### Mechanisms of drug resistance

Chemotherapy is the first-line approach to the treatment of cancers, whereas it seems to result in drug resistance and lead to complications. Therefore, exploring novel, safe, and highly effective treatments is imperative 80, 81. Cisplatin (CDDP) as a custom drug for treating cancers has usually been applied in clinical patients. However, decreased CDDP sensitivity in tumors becomes the biggest obstacle in cancer therapy 82. Recently, several studies have shown that miRNAs were associated with cancer drug resistance; for instance, it was reported that miR-134 plays various roles in drug resistance in diverse cancers 83. Furthermore, miR-128-3p was found to confer chemoresistance-associated metastasis in NSCLC 84. Similarly, miR-340 was also involved in cancer drug resistance. Forced miR-340 expression in drug-resistant OS cells significantly reduced multidrug resistance-associated protein 1 and P-gp expression, and enhanced their sensitivity to CDDP by targeting *ZEB1* and *LPAATβ* 85, 86. In HCC, miR-340 was significantly downregulated, whereas Nrf2 was upregulated in HepG2/ CDDP cells. Transfection of miR-340 mimicked the suppressed Nrf2-dependent antioxidant pathway, and enhanced the sensitivity of HepG2/CDDP cells to CDDP 87. Further investigation uncovered that the NRAL/miR-340-5p/Nrf2 axis mediated CDDP resistance in HCC 88. In addition, miR-340-5p was reported to be involved in trastuzumab resistance in HER2-positive BC cells 89. Melanoma is usually highly refractory to chemotherapy, which is mainly due to the high heterogeneity and plasticity of melanoma cells that is strictly connected to changes in tumor microenvironment 34. Wozniak et al. uncovered that the increased levels of ABCB5, a transmembrane transporter involved in drug resistance considered as a marker of melanoma stem-like cells, could be a result of a significant miR-340-5p downregulation 34.

Additionally, miR-340 was also involved in radiation-induced aggressive tumor formation 10. It has been reported that interleukin-4 (IL-4) and IL-4Rα (IL-4 receptor) were highly expressed in various human cancer cells following radiation treatment. High expression of IL-4 in patients with cancer is strongly correlated with poor survival. It has been found that IL-4 expression was reduced by miR-340 and miR-429, which were in turn downregulated by ionizing radiation. This study presented a conceptual advance suggesting that combining radiotherapy with genetic therapy to inhibit IL-4 may be a promising strategy for preventing post-radiation recurrence and metastasis in patients 10. Treatment with the natural compound curcumin or miR-340 induced pancreatic cancer cell apoptosis 36. Curcumin could increase miR-340 expression and reduce the expression of the oncogene *XIAP*, which was identified as a direct target of miR-340. This interaction may provide the basis for novel treatment strategies for patients with pancreatic cancer 36.

These findings suggest that miR-340 plays various roles in cancer drug resistance. A combination of genetic therapy with chemotherapy or radiotherapy may be a useful strategy to overcome drug resistance; however, this approach needs further investigation to validate it before applying it to clinical therapy.

## Potential clinical applications of miR-340 in cancers

### miR-340 serves as a promising biomarker

Cancers have often progressed to middle and advanced stages at the time of diagnosis; therefore, early tumor and carcinoma screening and diagnosis are crucial. Evidence suggests that miRNAs are stable and could not be easily degraded in patient serum 90. Therefore, miRNAs might be a class of novel biomarkers valuable for the detection of diseases. Pulmonary sarcoidosis is associated with dysregulated expression of intracellular miRNAs. Novosadova et al. revealed that the miR-340-5p levels were dysregulated in the serum of patients with LS, implying that miR-340 might play a diagnostic role 14. In glioma patient serum 91, 92, miR-340 was found to be significantly elevated, and further bioinformatics analysis found that it possibly played important roles in the regulation of glioma signaling pathways, suggesting that miR-340 of the peripheral blood might serve as a new biomarker for glioma diagnosis, with high specificity and sensitivity. In the analysis of global miRNA expression profile in GC, miR-340 was found to be highly elevated in cancer cells, which suggests a potential role of miR-340 in the diagnosis of GC 93. As we have detailed earlier, miR-340 was found to be dysregulated in a variety of human tumors (**Table 1**), which might make it a promising and qualified biomarker for cancer diagnosis.

### miR-340 in cancer patient survival and prognosis

With the mechanisms of the function and regulation of miRNAs in cancer gradually revealed, miRNA-based treatment might be a possible candidate approach in the near future. It has been reported that miR-340 was notably downregulated in NSCLC tissues, and lower miR-340 expression was positively correlated with lymph node metastasis, larger tumor size, advanced TNM stage, and poor prognosis in NSCLC patients 40. Therefore, miR-340 might be a promising tool for treating cancers or exploring mechanisms of disease progression. In BC, miR-340 was observed to be downregulated and loss of miR-340 expression was associated with shorter overall survival 70. Recent studies have shown that exosomes were detected as an indicator for diagnosis and prognosis of BC in clinical settings. miR-340-5p was identified to be associated with tumor recurrence or distant organ metastasis in BC patients through its detection in patient peripheral blood exosomes, which can be used as prognostic biomarker in these patients 7, 94. GBM generally has a survival rate of 12 months from diagnosis. Further investigation demonstrated that miR-340 may thus represent a novel marker for GBM diagnosis and prognosis, and may be developed into a tool to improve the treatment of GBM 95. Another study suggested that a miR-340-5p-macrophage feedback loop modulated the progression and tumor microenvironment of GBM, and may represent a prognostic biomarker 96.

DTCs in the bone marrow can be a sensitive marker for cancer spread from the primary tumor, which is associated with prognosis; EpCAM acts as a specific epithelial cell protein for detecting DTCs in the bone marrow in patients with CRC 97, 98. miR-340 expression was significantly decreased in EpCAM(+) bone marrow cells in patients with liver metastasis. Survival analysis in 136 patients with CRC indicated that lower miR-340 expression was correlated with shorter five-year disease-free survival and poorer five-year overall survival 98. In addition, the CRC group with low miR-340 and high c-Met expression had the worst prognosis 78. Consistently, Yang et al. revealed that CRC patients with a low expression of miR-340-5p had a shorter overall survival and progression-free survival (PFS) than those with a high expression of miR-340-5p 99.

This part of the paper has focused upon the relationship between miR-340 and patient survival and prognosis in some cancers. We hope it provides evidence for further studies on cancer patient prognosis as well as disease burden. ROCK1, a protein serine/threonine kinase, was reported to function as a key modulator of cell motility, tumor cell invasion, and actin cytoskeleton organization 100. Recently, ROCK1 was discovered to be a target of miR-340 and was dramatically upregulated in GBM tissues and cells. Survival in GBM patients with high levels of miR-340 was significantly extended in comparison to that in patients with low levels of miR-340, which suggested that miR-340 was a glioma killer and a potential prognostic biomarker and therapeutic target in GBM 52. A similar relationship between miR-340 and ROCK1 was revealed in pediatric OS 101. miR-340-low/ROCK1-high expression was significantly associated with both shortest overall survival and PFS. Further analysis suggested that the combined miR-340 downregulation and ROCK1 upregulation might be linked to tumor progression and adverse prognosis in pediatric OS 101. Yin et al., however, found that the expression of miR-340 was significantly elevated in both GC tissues and cells. Patients with high expression of miR-340 had shorter overall survival and disease-free survival 41. These data suggest that miR-340 may serve as a novel prognostic biomarker in GC. This conclusion is completely opposite to those of other investigations, whether there will be more analogous findings or not needs to be confirmed.

### Potential applications of miR-340 in cancer therapy

As the mechanism and role of miRNAs in human diseases are gradually unraveled, recent studies have started exploring the role of miRNAs as therapeutic agents. miR-340 acts as a tumor suppressor in multiple types of cancers, and emerging evidence has shown that miR-340-based united therapy was a promising approach to cancer treatment 17, 102. The intratumoral expression of miR-340 prior to neoadjuvant chemotherapy could be used to predict pathologic complete response (pCR) and a profile of miR-340^high^ identified patients who were unlikely to achieve pCR, and therefore might benefit from alternative treatment options, including earlier surgery 103. This study identified miRNAs as promising predictive biomarkers, which could aid in optimization of BC management and treatment stratification 103. Combined with high throughput sequencing analysis, miR-340 activity in cell proliferation, adhesion to the extracellular matrix, and tumor cell invasion, were identified in a tissue model, which was constructed by GBM developed into a stem cell-derived human engineered neural tissue, and also confirmed in GBM biopsies. miR-340 was a strong modulator of GBM aggressiveness and may constitute a therapeutic target for treatment of malignant gliomas 104. Similarly, restoration of miR-340 levels dramatically inhibited glioma cell proliferation, induced cell-cycle arrest and apoptosis, suppressed cell motility, and promoted autophagy and terminal differentiation, indicating an important role of miR-340 as a glioma killer, and suggesting a potential prognosis biomarker and therapeutic target for GBM 52. Takeyama et al. discovered that pre-miR-340 administration inhibited the growth of colon cancer cells and suppressed c-Met expression *in vitro* 78. Further analysis demonstrated that systemic pre-miR-340 administration suppressed the growth of pre-established HCT116 tumors in animal therapeutic models. These findings indicated that miR-340 may be useful as a therapeutic tool to treat CRC 78. Similarly, miR-340 may act as a potential new therapeutic target for the treatment of OS 77. A recent study revealed that the overexpression of miR-340 promoted macrophages to acquire M1-like phenotype polarized in peripheral and tumor immune microenvironments and increased T cell levels, especially the CD8^+^ T cells, contributing to the antitumor effect of miR-340 on pancreatic ductal adenocarcinoma 105. Taken together, our results indicate that miR-340 plays a role as an active agent against tumors, which merits further investigations for clinical development in cancer disease.

## Conclusions and future perspectives

In this review, we presented some interesting findings that might be beneficial for clinical applications and future studies. miR-340 is dysregulated in various tumors and carcinomas, where it may function as either a tumor suppressor or an oncogene. For instance, miR-340 was shown to be downregulated in two studies 18, 106, but was upregulated according to other work on GC 41, 107. Functionally, miR-340 was discovered to either repress or promote cancer cell proliferation and xenograft development and boost tumor cell apoptosis, migration, and metastasis, as well as benefit patient survival and prognosis. The molecular and modulated mechanisms of miRNA are extremely complicated and variable, and we found that miR-340 has diverse target genes and becomes involved in sophisticated signaling pathways when it functions in cancer 19, 56. There will be other targets and signaling pathways of miR-340 related to cancer, which might demand further investigations. Recently, long non-coding RNAs (lncRNAs) have emerged as crucial regulatory factors in diverse pathological processes, especially in tumorigenesis. Evidence has demonstrated that miR-340 was sponged by lncRNAs involved in tumor initiation and development 108-111, which may provide a new direction for an miR-340-based investigation of cancers.

Up to now, the role of miR-340 has been gradually illustrated in various cancers and other diseases. However, its functions in most malignant diseases are ambiguous. It might take a long time before it can be applied in clinical settings, similar to miR-34a or miR-122. However, encouragingly, increasing developing strategies are accelerating the investigation process, which brings us hope to overcome the present issues. Combined with current research results, in the future, the investigation of miR-340-based transgenic mice may be a promising direction to illustrate its functions more clearly under physiological conditions. In this paper, we summarized the role of dysregulation of miR-340 in a variety of malignant diseases, especially in cancers, highlighting the multiple roles of miR-340 in cancer initiation and progression.

## Figures and Tables

**Table 1:** miR-340 Dysregulates in Various Cancers and Their Target Genes

Cancer Type	Target gene	Reference
**miR-340 Downregulation**		
BC	LGR5	56
BC	——	7, 103
BC	ZEB1	29
BC	CTNNB1, c-MYC	71
BC	c-Met	70
CRC	ANXA3	99
CRC	RLIP76	50
CRC	——	78
Gallbladder carcinoma	NT5E	79
GBM	——	91, 92, 104
GBM	NRAS	95
GBM	CDK6, cyclin-D1, cyclin-D2	42
GBM	Bcl-w, Sox2	53
GBM	ROCK1	52
GC	SPP1	59
GC	——	18
HCC	DcR3	112
HCC	SKP2	47
HCC	JAK1	48
HCC	Nrf2	87
Laryngeal squamous cell carcinoma	EZH2	46
Melanoma	——	34, 113
NSCLC	ZNF503	114
NSCLC	CDK4	40
NSCLC	SKP2	44
OC	FHL2	20
OC	BAG3	67
OC	NF-κB1	66
OS	STAT3	115
OS	LPAATβ	86
OS	ROCK1	77, 101
Oral Squamous Cell Carcinoma	Glut1	116
Pancreatic cancer	BICD2	117
PCa	MDM2	57
Triple-negative breast cancer	——	118
**miR-340 Upregulation**		
GC	SOCS3	19
GC	cyclin G2	41
GC	——	93, 107
Thyroid cancer	BMP4	58

BC: breast cancer; CRC: colorectal cancer; GBM: glioblastoma multiforme; GC: gastric cancer; HCC: hepatocellular carcinoma; NSCLC: non-small cell lung cancer; OC: ovarian cancer; OS: osteosarcoma; PCa: Prostate cancer.

## Abbreviations

miRNAs: microRNAs
RISC: RNA-induced silencing complex
BC: breast cancer
OC: ovarian cancer
NSCLC: non-small cell lung cancer
OS: osteosarcoma
CRC: colorectal cancer
GC: gastric cancer
GIC: glioma-initiating cell
GBM: glioblastoma (multiforme)
ROCK1: Rho-associated protein kinase 1
ZEB1: zinc finger E-box-binding homeobox 1
HRE: hypoxia response element
RPE: retinal pigment epithelium
HCC: hepatocellular carcinoma
PCa: prostate cancer
EMT: epithelial-mesenchymal transition
TGF: transforming growth factor
HBV: hepatitis B virus
DTC: disseminated tumor cell
CDDP: cisplatin
pCR: pathologic complete response
lncRNA: long non-coding RNA
